# Surveillance data for human leishmaniasis indicate the need for a sustainable action plan for its management and control, Greece, 2004 to 2018

**DOI:** 10.2807/1560-7917.ES.2021.26.18.2000159

**Published:** 2021-05-06

**Authors:** Myrsini Tzani, Alicia Barrasa, Annita Vakali, Theano Georgakopoulou, Kassiani Mellou, Danai Pervanidou

**Affiliations:** 1 European Programme for Intervention Epidemiology Training (EPIET), European Centre for Disease Prevention and Control (ECDC), Stockholm, Sweden; 2 National Public Health Organisation (EODY), Athens, Greece; 3 Epiet (European Programme for Intervention Epidemiology Training) scientific coordinator, European Centre for Disease Prevention and Control (ECDC), Stockholm, Sweden; 4 These authors contributed equally to this article

**Keywords:** leishmaniasis, visceral, cutaneous, surveillance, domestic, imported, public health, one health

## Abstract

**Background:**

The World Health Organization (WHO) lists human leishmaniasis as a neglected tropical disease; it is not under surveillance at European level.

**Aim:**

We present surveillance data for visceral (VL) and cutaneous (CL) leishmaniasis for the period 2004 to 2018 in Greece to assess their public health importance.

**Methods:**

We extracted data from the mandatory notification system to analyse separately imported and domestic cases of VL and CL. A case was defined by clinical manifestations compatible with VL or CL and laboratory confirmation.

**Results:**

Between 2004 and 2018, 881 VL (862 domestic, 19 imported) and 58 CL cases (24 domestic, 34 imported) were recorded. The mean annual notification rate of domestic VL was 0.5 per 100,000 (range: 0.12–1.43/100,000) with a statistically significant increasing trend (p = 0.013). Cases were reported by all regions. The highest notification rate occurred in the age group 0–4 years (1.3/100,000). Overall 24% (164/680) of the cases were immunocompromised and their proportion increased after 2010 (p < 0.001). The mean annual notification rate of domestic CL was 0.05 per 100,000 (range: 0.01–0.19/100,000) with the highest rate in the age group 5–14 years (0.03/100,000). Cases were recorded in six of the 13 regions. Among 34 imported CL cases, 29 were foreign nationals.

**Conclusion:**

VL is endemic in Greece, with an increasing trend and a considerable burden of severe disease and young children being most affected. CL is rarely reported. A sustainable action plan is needed to reduce the burden of VL and prevent local transmission of CL.

## Introduction

Leishmaniasis is a parasitic disease caused by intracellular protozoan parasites of the genus *Leishmania* (Trypanosomatidae family); it is endemic in more than 98 countries worldwide [1]. Visceral (VL) and cutaneous (CL) leishmaniasis are the most common forms of the disease. VL causes a systemic disease characterised by fever, hepatosplenomegaly, anaemia and lymph node enlargement and may be fatal without appropriate treatment, while CL mainly causes skin ulcers and is considered a less severe form of the disease [2]. The incubation period for VL varies from 10 days up to nearly 3 years and for CL from 2 weeks to 3 years [3]. The natural route of transmission is a bite of blood-feeding phlebotomine sandflies; it may be zoonotic or anthroponotic, depending on the parasite species and the geographical location [4].

In the Mediterranean basin, four *Leishmania* species are found: *Leishmania infantum*, which is the most common species in this area, *Leishmania major,* found in North Africa and the Middle East, *Leishmania tropica,* with a limited presence in Europe, and *Leishmania donovani,* reported in Cyprus [5-7]. *Leishmania infantum* is responsible for VL and sporadic CL cases in humans. Domestic dogs are considered the principal reservoir hosts for this parasite since they efficiently replicate the protozoan parasite and are preferred hosts for the vector, phlebotomine sandflies [5,8].

According to the World Health Organization (WHO), leishmaniasis is one of the seven most important tropical diseases and represents a serious threat to global health, as it has a broad spectrum of clinical manifestations with a potentially fatal outcome [9]. Leishmaniasis is also among the top 10 neglected tropical diseases globally [10]. It is estimated that worldwide 700,000 to 1 million new cases and 26,000 to 65,000 deaths occur annually [11]. In the WHO European Region, Israel, Turkey, Turkmenistan and Uzbekistan are the countries most affected by CL. VL is found particularly in Albania, Georgia, Italy and Spain [12]. In the European Union (EU), the disease is not included in the list of the communicable diseases under surveillance [13], so the actual burden of the disease in unknown. However, there is available literature regarding the epidemiological situation and burden of domestic leishmaniasis in a number of European countries [14].

Based on historical medical descriptions in Greece, signs and symptoms resembling VL were first reported from the Greek island of Spetses in 1835. Until the 1940s, both forms of the disease were spread in many areas of Greece [15]. The use of DDT to control malaria during the Second World War decreased the frequency of mosquito and sandfly-transmitted diseases. However, in the past 40 years, the disease has re-emerged and is endemic in the country [5,16].

The objective of this article was to summarise the available surveillance data for domestic and imported VL and CL in Greece for the period from 2004 to 2018, in order to better understand their epidemiology and how these diseases have evolved throughout this 15-year period, and to assess their public health importance in the country.

## Methods

### Data sources

Leishmaniasis has been included in the mandatory notification system (MNS) of the Hellenic National Public Health Organisation (EODY) since 1998. In 2003, MNS was redesigned and since then, cases from all over the Greek territory have been notified through a more detailed reporting form on a weekly basis.

Cases should be reported by clinical doctors or microbiologists both to the regional/local public health authorities and to EODY. The notification form includes information on the form of the disease (VL or CL), demographic characteristics of the cases (age, sex, place of residence, nationality), clinical manifestations, hospitalisation, a number of exposures (travel to an endemic country during the last 12 months before symptom onset, immunocompromised status, dog ownership, presence of stray dogs and sandflies in the vicinity of the patients’ residence) and laboratory findings (parasitological confirmation, serology, other methods). Data on HIV status are not reported. In addition, the outcome at the time of notification is recorded; however, follow-up of reported cases regarding the final outcome of the disease is not foreseen.

Data entry takes place at EODY and all confirmed cases of leishmaniasis are recorded.

### Case definitions

According to EODY’s case definition, a confirmed VL case is an individual with at least one clinical manifestation compatible with the disease (persistent intermittent fever, splenomegaly, substantial weight loss, anaemia, lymph node enlargement) and laboratory confirmation by demonstration of the pathogen or detection of its nucleic acid in a clinical sample and/or detection of antigens or antibodies specific for the pathogen. A confirmed CL case is an individual with the appearance of skin lesions (nodular or ulcerative, usually on exposed areas of the body, which can be followed in some cases by the appearance of mucosal lesions) and laboratory parasitological confirmation (demonstration of the pathogen or detection of its nucleic acid in a clinical sample). In case only mucosal lesions are present, laboratory confirmation is performed via serology.

Imported cases were defined as: (i) VL cases of foreign nationality originating from an endemic country with symptom onset before arriving to Greece or in the first 2 weeks after arrival, (ii) CL cases with travel history abroad to an endemic country during the last 12 months before symptom onset, and (iii) CL cases of foreign nationality originating from a CL-endemic country with unknown/missing travel history.

Since VL is endemic in Greece, VL cases not falling into these categories, such as VL cases with travel history to an endemic country during the last 12 months before symptom onset for whom the country of exposure could not be defined, and VL cases of foreign nationality originating from an endemic country with unknown travel history, were analysed along with the domestic cases.

### Data analysis

Data were extracted from the MNS database for the period 2004 to 2018 and analysis was performed separately for VL and CL cases, as well as for imported and domestic cases.

We calculated the annual notification rate of domestic leishmaniasis (cases/100,000 population) for the period 2004 to 2018 using population data from the Hellenic Statistical Authority. In a negative binomial model using the monthly reported number of domestic VL cases between 2004 and 2018, we tested whether there was an increasing or decreasing trend in the notification rate of VL during the study period and whether there was seasonality. Incidence rate ratio (IRR) as a measure of association and 95% confidence intervals (95% CI) were calculated. Values of IRR ≥ 1 indicate an increasing trend. For domestic CL cases, this analysis could not be performed because of the small annual number of reported cases.

The mean annual notification rate per 100,000 population was calculated by region (Nomenclature of Territorial Units for Statistics (NUTS-2) of the cases’ residence for 2004–2018 for domestic VL and CL cases [17]. Maps were drawn using the European Centre for Disease Prevention and Control (ECDC) map maker tool (EMMA).

To describe the demographical characteristics of domestic VL and CL cases, we analysed the distribution by age group (0–4, 5–14, 15–24, 25–44, 45–64, ≥ 65 years), sex and nationality. Moreover, we calculated the proportion of cases by exposure.

Proportions of hospitalisation, clinical manifestations, immunocompromised status, diagnostic laboratory method used and proportion of cases with available typing results were calculated for the total (both domestic and imported) recorded cases of VL and CL.

Finally, in order to check if immunocompromised status has changed over time, we applied a chi-squared test to compare the proportion of immunocompromised among domestic VL cases between the periods 2004 to 2010 and 2011 to 2018.

P values < 0.05 were considered statistically significant. Data were analysed using Stata v16.1.

## Results

Between 2004 and 2018, 881 VL and 58 CL cases were reported. Overall, a total of 886 (94%) cases were classified as domestic (862 VL and 24 CL) and 53 (6%) as imported (19 VL and 34 CL). The 886 domestic cases include 76 cases who did not fulfil the criteria to be characterised either as imported or as domestic cases and were therefore analysed along with the domestic cases.

### Visceral leishmaniasis cases

#### Domestic cases of visceral leishmaniasis

**Figure 1:**
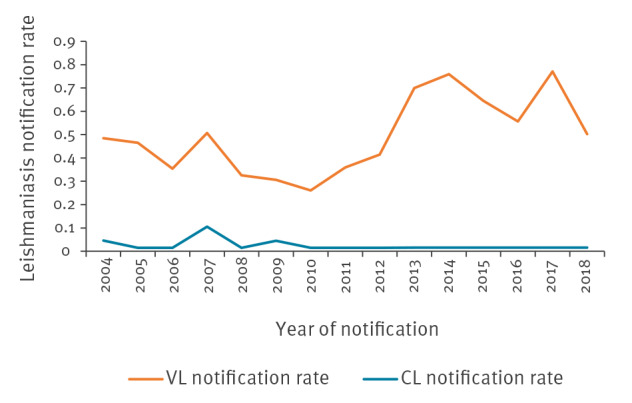
Annual notification rate (cases/100,000 population) by year of notification of visceral and cutaneous domestic leishmaniasis cases, Greece, 2004–2018 (n = 886)

CL: cutaneous leishmaniasis; VL: visceral leishmaniasis.

The mean annual notification rate of domestic VL at country level during the study period was 0.49 per 100,000 (range: 0.12–1.43/100,000) with a statistically significant increasing trend over time (p = 0.013) (Figure 1). There was no seasonal change in the notification rate of the disease.

**Figure 2:**
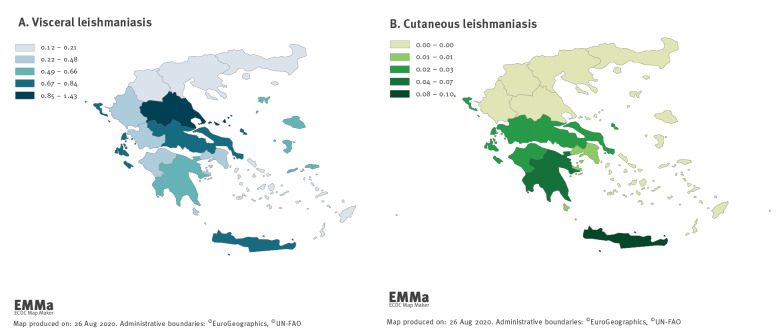
Mean annual notification rate (cases/100,000 population) for visceral and cutaneous domestic leishmaniasis cases by region, Greece, 2004–2018 (n = 886)

VL cases were recorded in all 13 regions of Greece. Figure 2 depicts the mean annual notification rate of VL cases by region.

Among the domestic VL cases with available information on sex, 65% (556/860) were male. The mean annual notification rate was 0.70 per 100,000 among male and 0.37 per 100,000 among female cases. The median age of domestic VL cases was 42 years (range: 0–87) for male and 39 years (range: 0–90) for female cases.

**Figure 3:**
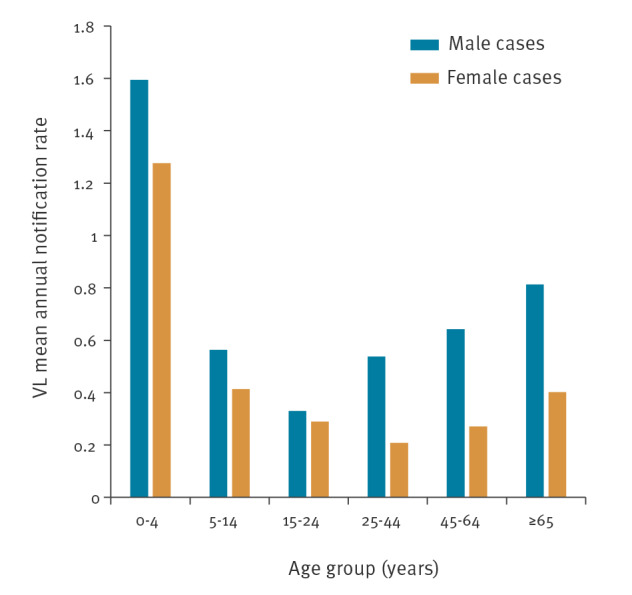
Mean annual notification rate of visceral domestic leishmaniasis, by age group and sex, Greece, 2004–2018 (n = 860)

The mean annual notification rate of VL by sex and age group is presented in Figure 3. The age group 0–4-years-old was the most affected for both male and female cases.

VL: visceral leishmaniasis.

Ninety per cent (770/860) of domestic VL cases were of Greek nationality; among the patients with foreign nationality, 74% (59/80) were Albanians. The rest of the cases originated from 13 different countries, seven European and six non-European.

Among domestic VL cases for whom the respective information was available, 35% (259/738) owned a dog with no canine leishmaniasis diagnosis and 1% (11/738) with canine leishmaniasis. Among cases for whom the respective information was available, 563 of 732 (77%) reported the presence of stray dogs and 533 of 697 (76%) the presence of sandflies in the vicinity of their residence.

Persistent fever (86%; 657/760) and hepato- or splenomegaly (83%; 631/760) were the most commonly reported clinical manifestations of VL cases. Lymph node enlargement was reported in 16% (122/760) of cases and skin and/or mucosal lesions were reported in 3% (25/760) of cases, while 96% (827/862) of all cases were hospitalised and 24% (164/680) were immunocompromised.

For cases with available information, the proportion of immunocompromised cases among domestic VL cases was 9% (32/349) and 25% (126/513) for the periods 2004 to 2010 and 2011 to 2018, respectively. The difference between the two proportions was statistically significant (p < 0.001).

Overall, for 633 cases with known information, the median time interval between symptom onset and hospitalisation was 14 days (range: 0–380). Among 339 cases with known definite outcome at the time of notification, 14 (4%) fatal cases and 325 (96%) cured cases were recorded; however, for almost half, the outcome was not definite at the time of notification. For five of the 14 fatal cases, immunocompromised status was reported.

Serological, parasitological and PCR testing was positive in 93% (555/600), 93% (336/360) and 99% (277/279) of the tested cases, respectively. The type of VL pathogen was reported in 7% (67/939) of the total recorded cases. Of the 67 VL cases, 39 were typed as *Leishmania donovani* complex (without any further typing) and the remaining 28 as *L. infantum.*

#### Imported cases of visceral leishmaniasis

**Figure 4:**
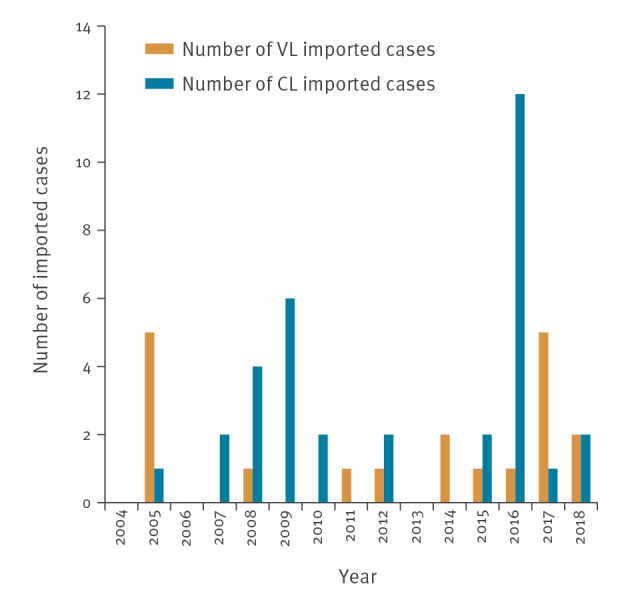
Number of imported visceral and cutaneous leishmaniasis cases, Greece, 2004–2018 (n = 53)

CL: cutaneous leishmaniasis; VL: visceral leishmaniasis.

Among the 19 imported VL cases, 15 were male and the median age of cases was 24 years (range: 2–76). All cases were of foreign nationality, mainly from Albania (13/19); the remaining six were from four different countries (Iraq, Libya, Russia and Syria). Twelve of the 13 Albanians with available information on travel history had been travelling for the purpose of visiting friends and relatives. Figure 4 presents the number of imported VL cases by year.

### Cutaneous leishmaniasis cases

#### Domestic cases of cutaneous leishmaniasis

The mean annual notification rate of domestic CL at country level for the years 2004 to 2018 was 0.05 per 100,000 (range: 0.01–0.19/100,000) (Figure 1). Figure 2 shows the mean annual notification rate of CL cases by region. Domestic CL cases were recorded in six of the 13 Greek regions, with the majority of cases recorded in the southern part of the country, and Crete.

**Figure 5:**
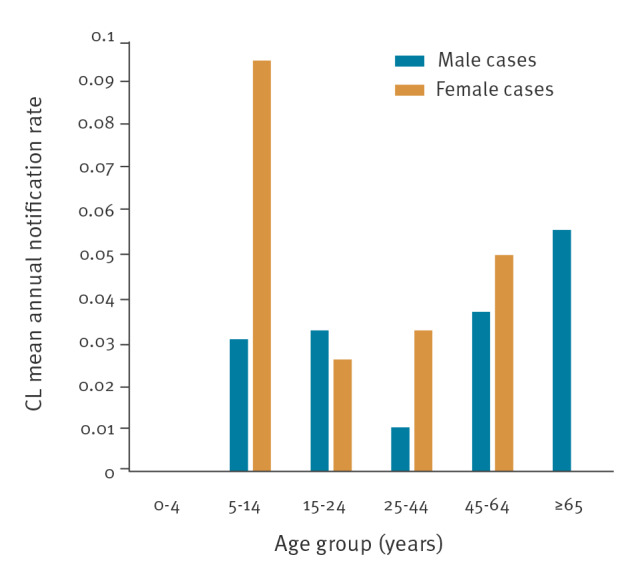
Mean annual notification rate of domestic cutaneous leishmaniasis cases, by age group and sex, Greece, 2004–2018 (n = 24)

Among the domestic CL cases, 10 were male and the median age was 58 years (range: 5–88). The mean annual notification rate of CL by sex and age group is presented in Figure 5. The age group 5–14 years was the most affected among the female cases and the age group older than 65 years was the most affected among the male cases.

CL: cutaneous leishmaniasis.

Twenty-three of the 24 domestic CL cases were of Greek nationality. Among those for whom this information was available, one of 13 owned a dog with no canine leishmaniasis diagnosis, 10 of 14 reported the presence of stray dogs and nine of 13 reported the presence of sandflies in the proximity of the patient’s residence.

Among the total reported CL cases, nodular (28/46) and ulcerative skin lesions (22/46) were the most commonly recorded clinical manifestations and three of 46 had mucosal lesions. Forty-three of the 56 cases were hospitalised and one of 31 reported underlying immunosuppression.

For 20 cases with known information, the median time interval between symptom onset and hospitalisation was 47 days (range: 0–435). No fatalities were reported among the 16 cases with recorded definite outcome at the time of notification.

Parasitological testing was positive in all 35 of the cases tested. Similarly, PCR was positive in all 13 of the cases tested, whereas a positive serological test was reported in eight of 10 tested cases. In 0.4% (4/939) of the total recorded cases, the type of CL was reported. Among these four CL cases, two were typed as *L. infantum,* one as *L. donovani* complex (without any further typing) and one as *L. tropica.*

#### Imported cases of cutaneous leishmaniasis

Among the imported CL cases, 22 were male and the median age of the cases was 24.5 years (range: 2–76).

Most imported CL cases (29/34) were of foreign nationality, mainly refugees/immigrants from Syria (14/29), Afghanistan 7/29) and Pakistan (4/29). The largest number (12/34) of CL imported cases were recorded in 2016. Figure 4 presents the number of imported CL cases by year.

## Discussion

This 15-year analysis of the MNS’s epidemiological data for leishmaniasis in Greece is the first after the analysis performed by Gkolfinopoulou et al. for the period 1981 to 2011 [16].

VL continues to be endemic in Greece as domestic cases are recorded every year across the Greek territory. The disease is also endemic in a number of other southern European countries and cases have been recorded in neighbouring countries such as Bulgaria, Turkey, North Macedonia and Albania [1,18]. Imported VL cases in Greece constitute a small proportion of the total reported cases.

From 2004 to 2018, the notification rate of domestic VL increased. The reported mean annual notification rate of VL in Greece for the period 2004 to 2018 was 0.49 per 100,000 population. This is in line with the respective estimates in southern Europe (0.02–0.49/100,000 population) [18]. However, we can consider that the true incidence of VL is higher, taking into consideration the under-reporting rate in Greece which has been crudely estimated by the WHO to range between 1.2–1.8 fold [16]. Furthermore, because the estimated prevalence of asymptomatic human carriers of *L. infantum* in southern Europe is high, e.g. 20.7% in the south-western Madrid region and 21% in Monaco, it is difficult to estimate the true leishmaniasis infection rate, as only symptomatic cases are recorded at MNS [19,20].

A considerable proportion of VL cases were immunocompromised and more than one third of fatal VL cases were immunocompromised. An increase in co-infections with human immunodeficiency virus (HIV) and *Leishmania* that has been observed since the 1980s, resulted in leishmaniasis becoming the third most frequent opportunistic parasitic disease after toxoplasmosis and cryptosporidiosis in Europe [21]. Moreover, the continuous increase in the proportion of immunocompromised people in the population as well as the increasing use of anti-tumour necrosis factor therapies to target components of the immune response that are also key in controlling infection with leishmaniasis could result in a higher burden of the disease in the future [22,23]. According to the epidemiological data available at MNS, the proportion of immunocompromised among domestic VL cases in Greece increased during the study period.

Unlike VL, CL is rarely reported in Greece. This can probably be attributed to the fact that CL can frequently manifest in a very benign form and therefore remains unreported [24]. From 2004 to 2018, the number of reported CL cases did not differ significantly over time, and most of the cases were imported. The number of imported CL cases peaked in 2016 because of the increase in the number of refugees arriving from Syria and Afghanistan [25].

Geographical differences in the notification rates for both forms of the disease within Greece are evident. Possible contributing factors could include differences in the local climate, abundance of competent vectors and reservoir hosts, as well as differences in case detection and reporting rates. Geographical differences between regions within the same country have also been reported by other EU countries [14].

Regarding the age distribution of domestic VL cases, young children (age group 0–4 years) were the most affected, whereas for CL, the age group 5–14 years presented the highest mean annual notification rate. These results are consistent with those in other Mediterranean countries, where the incidence/notification rate of leishmaniasis is highest among children and childhood VL infection may account for more than half of all reported cases [26]. According to the literature, young age is a risk factor for developing clinical illness [26]. For adults, the majority of VL and CL cases were male, probably reflecting to some degree their higher exposure to vectors during outdoor agricultural work or other outdoor activities. The same has been also reported for other vector-borne diseases [27].

VL and CL domestic cases were mostly of Greek nationality, followed by cases originating from Albania. This was not unexpected, given the large number of Albanians residing permanently in Greece; according to the Hellenic Statistical Authority, Albanians constitute 52.7% of the immigrants in the country (almost 480,000 people) [28]. In addition, Albanians constituted the majority of the imported VL cases after travelling to their country of origin which is also endemic for VL [1].

The proportion of hospitalised cases was high for both VL and CL; for VL, this could be explained by the fact that clinical cases usually require hospitalisation for the case management, and for CL, the more severe cases are probably hospitalised and thus diagnosed and reported [24].

The majority of VL and CL cases reported presence of stray dogs in the proximity of their residence. Official data on the number of stray dogs in the country are not available. Around 10% of dogs in endemic countries are seropositive for *L. infantum*, with wide variations between regions [8]. PCR studies in endemic areas have indicated much higher prevalence than serology, with up to 80% of the dog population testing positive [8]. This emphasises importance of systematically controlling the population of stray dogs in the country as well as controlling the infection in the dog population, by improving canine leishmaniasis surveillance, promoting dog vaccination against canine VL, protection from sandfly bites, regular testing and implementation of proper treatment and management of infected dogs [8].

The presence of sandflies, which are the main vectors of the disease, was reported by a large proportion of both VL and CL cases in proximity with their residence. This was also expected, as climate conditions in Greece are favourable for vector development [29]. *Phlebotomus neglectus*, *P. tobbi* and *P. perfiliewi* are the vector species which transmit *L. infantum*, the species responsible for the VL and some of the CL cases in Greece, while *P. sergenti* is the vector competent for transmitting *L. tropica,* which has caused anthroponotic CL cases in the country [16]. A recent study performed in Greece detected high *L. donovani* and *L. tropica* infection rates of *Phlebotomus* spp. sandflies collected from refugee camps, indicating a potential risk of local anthroponotic transmission and of potential introduction of new *Leishmania* species [30]. The natural vector of *L. tropica*, which causes anthroponotic CL, exists in southern Europe (and a few sporadic cases were recorded in the past in some areas in Greece), posing a probable risk of further spread after introduction through imported cases into Greece and other countries with competent vectors [17]. This demonstrates the need for enhanced surveillance and improvement of typing diagnostic capacity in the country.

The main limitations in the surveillance of leishmaniasis in Greece were the limited information about disease outcome and the lack of systematic typing, which makes it difficult to detect the introduction of new species in a timely manner, to distinguish the different species of CL in the country and thus to differentiate the endemic from sporadic cases [31]. Because Greece is an endemic country for leishmaniasis, there were 76 cases for whom it could not be precisely determined whether they were domestic or imported; we analysed these cases together with the domestic ones, which probably resulted in an overestimation of the domestic cases.

## Conclusion

Leishmaniasis should be a public health priority in Greece because the visceral form of the disease is endemic in almost all areas of the country, it causes severe symptoms and mostly affects young children. In addition, there is a risk of introduction of new *Leishmania* species, especially in connection with migration from endemic countries. Therefore, a sustainable action plan for leishmaniasis prevention and control in the context of a One Health approach should be developed, including enhanced surveillance and laboratory capacity, reservoir and vector control, timely diagnosis and treatment of cases as well as health education and communication campaigns for the public.
